# FOXM1 mediates methotrexate resistance in osteosarcoma cells by promoting autophagy: The role of FOXM1 in drug resistance in osteosarcoma

**DOI:** 10.3724/abbs.2024084

**Published:** 2024-07-31

**Authors:** Luoyang Wang, Dongchang Zhai, Lei Tang, Hui Zhang, Xinlong Wang, Ning Ma, Xiaoyue Zhang, Mingguo Cheng, Ruowu Shen

**Affiliations:** 1 Department of Immunology School of Basic Medicine Qingdao University Qingdao 266071 China; 2 Department of Special Medicine School of Basic Medicine Qingdao University Qingdao 266071 China; 3 Organ Transplantation Center the Affiliated Hospital of Qingdao University No.59 Haier Road Laoshan District Qingdao 266061 China; 4 Department of Basic Medicine School of Basic Medicine Qingdao University Qingdao 266071 China; 5 Department of Medicine School of Basic Medicine Qingdao University Qingdao 266071 China; 6 Orthopedic Surgery The Third People’s Hospital of Qingdao Qingdao 266100 China

**Keywords:** osteosarcoma, drug resistance, FOXM1, methotrexate

## Abstract

Osteosarcoma (OS) is a primary bone cancer mostly found in adolescents and elderly individuals. The treatment of OS is still largely dependent on traditional chemotherapy. However, the high incidence of drug resistance remains one of the greatest impediments to limiting improvements in OS treatment. Recent findings have indicated that the transcription factor FOXM1 plays an important role in various cancer-related events, especially drug resistance. However, the possible role of FOXM1 in the resistance of OS to methotrexate (MTX) remains to be explored. Here, we find that FOXM1, which confers resistance to MTX, is highly expressed in OS tissues and MTX-resistant cells. FOXM1 overexpression promotes MTX resistance by enhancing autophagy in an HMMR/ATG7-dependent manner. Importantly, silencing of *FOXM1* or inhibiting autophagy reverses drug resistance. These findings demonstrate a new mechanism for FOXM1-induced MTX resistance and provide a promising target for improving OS chemotherapy outcomes.

## Introduction

Osteosarcoma (OS) is a rare primary bone cancer that is mostly diagnosed in adolescents and elderly individuals [1]. OS can occur in any bone in the body [2]. The age-adjusted incidence of OS in children and adolescents aged 0 to 24 years is 4.4 per million people annually [3]. Children aged 0 to 9 years have the best overall 5-year relative survival rate (71.8%), followed by adolescents aged 10 to 24 years (65.9%) [1]. Elderly individuals >60 years of age have the worst overall 5-year relative survival rate, at 33.1% [1]. OS is a highly fatal disease due to the high incidence of pulmonary metastases until systemic chemotherapy is combined with surgery [4]. Doxorubicin, methotrexate (MTX), cisplatin, and ifosfamide have been suggested to be the most efficacious agents for treating OS. These drugs are commonly applied in combination with at least three of them to achieve the best outcomes [4]. However, it has been revealed that adding more agents to the treatment regimen does not lead to further benefits [5].

In addition, drug resistance is a major hurdle that severely limits the improvement of OS treatment [6]. The antifolate drug MTX can promote apoptosis mainly by binding to the dihydrofolate reductase enzyme to inhibit DNA synthesis and replication and is widely used to treat rheumatoid arthritis and many cancers, including OS. However, OS may acquire resistance to MTX treatment through a variety of mechanisms, such as increased expression of dihydrofolate reductase enzyme, the folate carrier SLC19A1, and ABC transporters [6]. Identifying novel drug resistance mechanisms may provide new therapeutic targets and further improve OS chemotherapy outcomes.

FOXM1, a member of the forkhead box protein family, is a crucial transcription factor that regulates multiple activities of cancer cells, such as growth, metastasis, recurrence, and stem cell features [7]. Recent findings have indicated the importance of FOXM1 in the modulation of chemotherapeutic resistance in many cancers [ 8– 11] . FOXM1 expression is significantly increased in osteosarcoma [ 12, 13] , and FOXM1 decreases tumor formation [14], proliferation, migration, and invasion [13]. Moreover, FOXM1 can induce chemoresistance to cisplatin treatment in OS by activating the expression of multidrug resistance protein 1 [15]. Downregulating FOXM1 expression enhances the sensitivity of OS to cisplatin [16]. FOXM1 is emerging as an attractive target for overcoming chemotherapeutic drug resistance [17]. However, whether FOXM1 participates in the development of MTX resistance in OS remains to be investigated.

In this study, we confirmed that FOXM1 is highly expressed in OS tissues and FOXM1 overexpression contributes to drug resistance in MTX-resistant OS cells. Silencing of *FOXM1* enhanced the sensitivity of OS cells to MTX. Mechanistically, we found that FOXM1 could increase the level of autophagy and protect OS cells from apoptosis after treatment with MTX by inducing the expression of the hyaluronan-mediated motility receptor (HMMR). These findings reveal a critical role for FOXM1 in OS resistance to MTX and provide a promising target to increase the sensitivity of OS to MTX.

## Materials and Methods

### Cell culture and treatment

U-2OS and MG-63 human osteosarcoma cell lines and human normal osteoblast cell line (hFOB1.19) were purchased from Procell Life Science & Technology (Wuhan, China). 143B osteosarcoma cell line was a kind gift from Dr. Bin Yue of the Department of Bone Oncology, the Affiliated Hospital of Qingdao University. These cells were maintained at 37°C with 5% CO _2_ in high-glucose DMEM containing 10% fetal bovine serum (BioInd, Beit Haemek, Israel). MTX-resistant OS cells were established by exposing parental OS cells to increasing concentrations of MTX as previously described [18]. Briefly, the cells were incubated with a gradually increasing concentration of methotrexate, U-2OS, starting at 0.1 μM and maintained until the methotrexate-sensitive cells died. The surviving cells were refilled with methotrexate, and after 3 months, the cells that divided freely in 100 μM methotrexate-containing medium were considered resistant cell lines and labeled “U-2OS/MTX”.

U-2OS and 143B cells of logarithmic growth stage were inoculated on 6-well plates. After adhesion, the cells were pretreated with or without Chloroquine (TargetMol, Shanghai, China) (20 μM) for 2 h and then treated with methotrexate (Sigma Aldrich, St Louis, USA) (50 μM) for 24 h.

### Tumor tissue collection

Paraffin sections of tumor tissues were obtained from The Affiliated Hospital of Qingdao University. Informed consent was obtained from all human subjects, and the use of human samples for immunohistochemistry was approved by the Experimental Ethics Committee of Qingdao University (No. QDU-HEC-2022005).

### Immunohistochemistry analysis

The sections were immobilized using 4% paraformaldehyde and permeabilized for 20 min with 0.5% Triton X-100. Next, the sections were blocked with 5% BSA for 1 h at room temperature and incubated with primary anti-FOXM1 (1:100; sc-271746; Santa Cruz) and overnight at 4°C. Finally, the sections were incubated with secondary Abs (1:500; HUABIO, Wuhan, China) for 1 h at 37°C. Cell nuclei were stained with DAPI (Beyotime, Shanghai, China). All images were collected by a BX50 fluorescence microscope (Olympus, Tokyo, Japan).

### Bioinformatics analysis

GSE16089 gene expression microarray data were downloaded from the GEO database ( https://www.ncbi.nlm.nih.gov/geo/). This dataset contained 3 samples of Saos-2 osteosarcoma cells sensitive to MTX and 3 samples of Saos-2 cells resistant to MTX. Student’s *t*-test was used for statistical analysis. TARGET database ( https://ocg.cancer.gov/programs/target), which contains both gene expression profiles and clinical information for patients with osteosarcomas, was used for survival analysis.

### Lentivirus and transfection

The negative control (NC) and FOXM1 overexpression or knockdown lentivirus were designed and synthesized by Genechem (Shanghai, China). The FOXM1 overexpression plasmid was constructed by subcloning FOXM1 cDNA into a GV492 plasmid (Ubi-MCS-3FLAG-CBh-gcGFP-IRES-puromycin). For gene silencing, the shRNA targeting FOXM1 mRNA was also cloned and inserted into the GV492 plasmid (U6-MCS-CBh-gcGFP-IRES-puromycin). The titer of the concentrated viral particles was 5×10 ^8^~1×10 ^9^. OS cells were seeded into 24-well plates and then inoculated with lentiviral particles at a multiplicity of infection (MOI) of 10. Puromycin (0.01 μM) was added to the cells for clone selection after infection for 24 h. The sh-FOXM1 gene sequence was 5′-CAGCTGGGATCAAGATTATTA-3′. The sh-NC sequence was 5′-UUCUCCGAACGUGUCACGUTT-3′. The Lv-FOXM1 gene sequence was 5′-CGAAAGATGAGTTCTGATGGACT-3′.

### Cell viability assay

OS cells were seeded in a 96-well plate at a density of 4000 cells per well. After treatment with MTX for the indicated time, the culture supernatant was discarded, 90 μL of culture medium and 10 μL of CCK-8 solution (TargetMol, Shanghai, China) were added to each well and incubated for 4 h at 37°C. The absorbance was measured at 450 nm using an automatic microplate reader (BioTek, Winooski, USA).

### Cell apoptosis analysis

Cell apoptosis was determined with an Annexin V-PE/7-AAD kit (Vazyme, Nanjing, China) according to the manufacturer’s protocol. Briefly, after treatment with MTX for 24 h, the cells were collected and resuspended in binding buffer at a final concentration of 1×10 ^6^ cells/mL. The cells were stained with 5 μL of Annexin V-PE and 5 μL of 7-AAD for 15 min at room temperature in the dark. The percentage of apoptotic cells was measured using a NovoCyte 2060 flow cytometer (Acea Biosciences, San Diego, USA) and analyzed with FlowJo software (Tree Star, Ashland, USA).

### Western blot analysis

Total proteins were extracted using RIPA lysis buffer (Strong) (CoWin Biotech, Taizhou, China), and the protein concentration was measured using a BCA protein assay kit (CoWin Biotech). A total of 20 μg of protein from each sample was separated by 10% SDS-PAGE and transferred to a PVDF membrane. The membranes were incubated in blocking buffer (TBST solution with 5% skim milk) for 1 h at room temperature. After being washed with TBST buffer, the membranes were incubated with primary and secondary antibodies. The proteins in the membranes were then detected with an enhanced chemiluminescence (ECL) kit (Yeasen, Shanghai, China) and imaged with a GE AI680 electrophoretic gel imaging analysis system. The antibodies against ATG7 (12741), FOXM1 (8558), LC3B (12741) were purchased from Cell Signaling Technology (Beverly, USA). The antibody against HMMR (15820-1-AP) was purchased from Proteintech (Wuhan, China). The antibodies against Bax (ER0907), and Cleaved-Caspase3-p17 (HA722367) were purchased from HUABIO (Wuhan, China).

### Immunofluorescence staining

Cells grown on cover glass slides were fixed with methanol for 15 min, washed, and blocked with 1% BSA for 1 h at room temperature. The cells were then incubated with an anti-LC3B antibody (Cell Signaling Technology, Beverly, USA) overnight at 4°C and with a TRITC-conjugated secondary antibody (Boster, Wuhan, China) for 1 h at room temperature. The slides were visualized using the BX50 fluorescence microscope.

### RT-qPCR

Total RNA was extracted from the cells with RNAiso Plus (Takara, Shiga, Japan) according to the manufacturer’s instructions. A total of 1 μg of RNA was transcribed into cDNA using Hifair III SuperMix plus (Yeasen). RT-qPCR was performed using a QuantStudio 3 Real-Time PCR System (Applied Biosystems, Foster City, USA) with qPCR SYBR Green Master Mix (Yeasen) under the following cycling conditions: 95°C for 5 min and 40 cycles of 95°C for 10 s and 60°C for 30 s. The relative fold change in gene expression was normalized to that of *β-actin* using the 2 ^–ΔΔCt^ method. The PCR primers used were as follows: 5′-GACATTGGACCAGGTGTTTAAGC-3′ (forward) and 5′-GGAAGCAAAGGAGAAAACCCTTC-3′ (reverse) for *FOXM1*, and 5′-TCCTGTGGCATCCACGAAACT-3′ (forward) and 5′-GAAG CATTTGCGGTGGACGAT-3′ (reverse) for *β-actin*.

### Coimmunoprecipitation assay

Total proteins were extracted from cells using RIPA lysis buffer (Strong), incubated with an anti-FOXM1 antibody (1:100; Cell Signaling Technology) and precipitated using Protein A/G magnetic beads (Yeasen). The beads were harvested, washed, and analyzed by western blot analysis.

### Animals and treatment

The animal experiment protocols were approved by the Experimental Animal Ethics Committee of Qingdao University (No. QDU-AEC-2021131). Balb/c nude mice (6 weeks old) were purchased from Vital River Laboratory Animal Technology (Beijing, China). All mice were maintained under specific pathogen-free conditions according to the guidelines of the Animal Care and Use Committee of Qingdao University. Briefly, 2×10 ^6^ sh-FOXM1- or sh-NC-transfected 143B cells (100 μL) were subcutaneously injected into the right flank of the mice. Tumor volumes were measured every 3 days with an electronic caliper and reported as a volume using the formula (width ^2^×length)/2. When the size of the xenografts in the mice reached 50 mm ^3^, the mice were treated with or without MTX (20 mg/kg body weight) by intraperitoneal injection every three days. Twenty-one days later, the mice were euthanized, and the tumors were weighed, and subject to subsequent analyses.

A TUNEL apoptosis Assay kit (Beyotime) was used to detect the apoptosis of cells in the tumor tissues of each group according to the manufacturer’s instructions. TUNEL-positive cells were stained with DAPI, five visual fields were randomly selected for observation, and the apoptotic cells were counted under the BX50 fluorescence microscope.

The level of LC3B protein in the tumor tissues was detected by immunofluorescence staining. Briefly, the tumor tissues were fixed in 4% paraformaldehyde at 4°C for 30 min, dehydrated with different concentrations of alcohol, infiltrated with xylene, embedded in paraffin, and sliced into 5-μm-thick sections. The slices were permeabilized with 0.3% Triton X-100 in PBS for 15 min and blocked with 3% bovine serum albumin for 30 min. The blocked sections were incubated with primary antibodies against LC3B (1:500; Cell Signaling Technology) overnight at 4°C and then with rabbit anti-mouse FITC secondary antibody (1:1000) for 1 h at room temperature. Nuclear staining with DAPI was performed after secondary antibody incubation. The slides were subsequently imaged under the BX50 fluorescence microscope.

### Statistical analysis

Statistical analysis was performed using GraphPad Prism 8.0 (GraphPad Software, La Jolla, USA). Data are presented as the mean±SEM. Student’s *t* test was used for comparisons between two groups, while one-way analysis of variance was used for comparisons among multiple groups. A difference was considered statistically significant if the *P* value was less than 0.05.

## Results

### FOXM1 is associated with MTX resistance in OS

**Figure 1:**
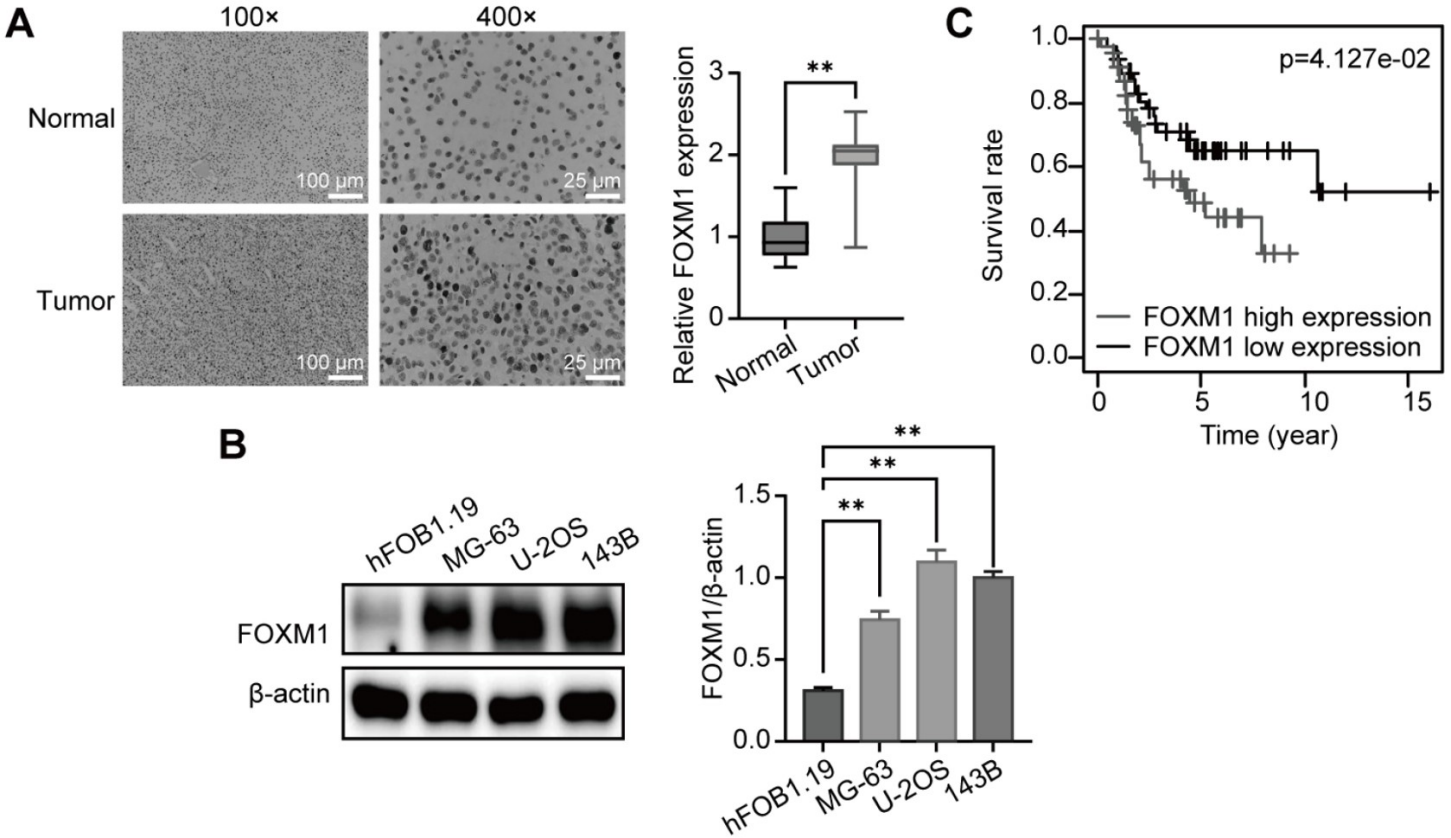
Figure 1 FOXM1 is highly expressed in OS and is associated with poor prognosis (A) Representative immunohistochemistry images and quantitative evaluation of the expression of FOXM1 in the tissues of OS patients. n=35. Scale bar: 100 or 25 μm. (B) Western blot analysis of FOXM1 expression in OS cells; β-actin was used as the loading control. n=3. (C) Kaplan-Meier curves for survival analysis of OS patients from the TARGET database. ** P<0.01.

We first analyzed the expression of FOXM1 in the tumor tissues of OS patients by immunohistochemistry. As shown in Figure 1A, FOXM1 expression was significantly greater in OS tumor tissues than in paracancerous tissues. We also compared the expression of FOXM1 in hFOB1.19 normal osteoblasts and OS cells. Western blot analyses confirmed that FOXM1 was more highly expressed in OS cells than in normal osteoblasts ( Figure 1B). We also examined the association between FOXM1 mRNA expression (and the survival rate of OS patients in the Therapeutically Applicable Research to Generate Effective Treatments (TARGET) database, and the results showed that high FOXM1 mRNA expression is associated with poor OS ( Figure 1C). These findings suggest the important role of FOXM1 in OS.

**Figure 2:**
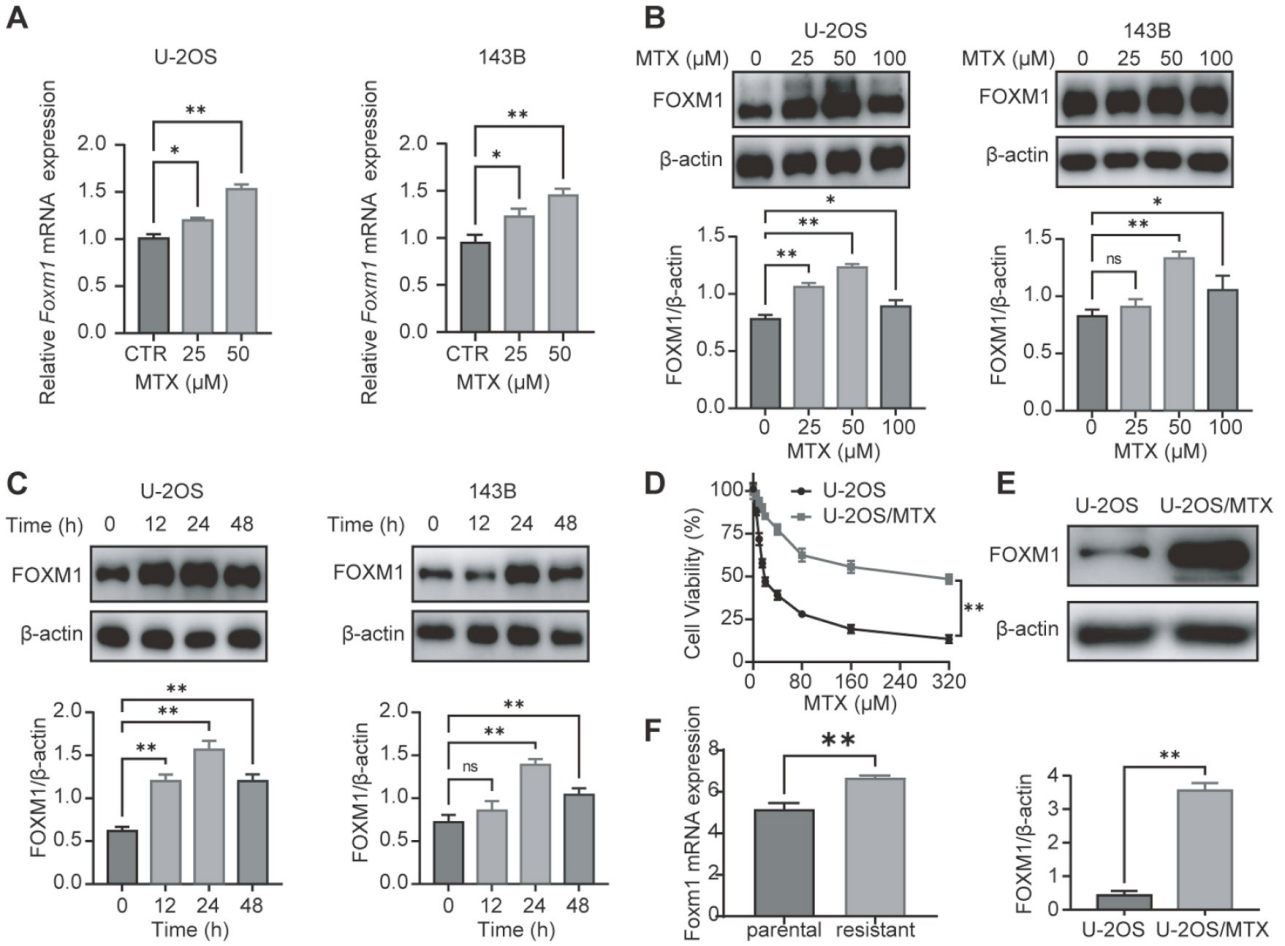
Figure 2 FOXM1 is overexpressed in MTX-resistant OS cells (A) After treatment with various concentrations of MTX for 24 h, FoxM1 mRNA levels in OS cells were estimated by qRT-PCR. (B,C) After treatment with different concentrations of MTX for 24 h or 50 μM MTX for different durations, FOXM1 protein levels in OS cells were detected by western blot analysis, and β-actin was used as the loading control. (D) Cell viability assay of both MTX-sensitive and MTX-resistant OS cells using CCK-8 after 24 h of incubation with MTX at different doses. (E) FOXM1 protein levels were detected by western blot analysis, and β-actin was used as the loading control. (F) FoxM1 mRNA levels in parental and MTX-resistant OS cells. n=3, * P<0.05, ** P<0.01.

To investigate the possible role of FOXM1 in MTX resistance in OS, we examined the expression of FOXM1 in OS cells at both the transcript and protein levels after treatment with MTX. As shown in Figure 2A–C, MTX treatment significantly increased the expression of FOXM1 in both time- and concentration-dependent manners. Since MTX at a concentration of 100 μM and treatment for 48 h showed certain drug toxicity, our subsequent study selected an optimal MTX concentration of 50 μM and an optimal time interval of 24 h. Furthermore, MTX-resistant OS cells (U-2OS/MTX) were established by intermittently exposing U-2OS cells to gradually increasing concentrations of MTX ( Figure 2D). Western blot analyses confirmed that FOXM1 was significantly overexpressed in MTX-resistant OS cells ( Figure 2E). Additionally, similar results were obtained in the GEO dataset GSE16089 ( Figure 2F). These data indicate that FOXM1 is associated with MTX resistance in OS.

### FOXM1 overexpression mediates MTX resistance in OS

**Figure 3:**
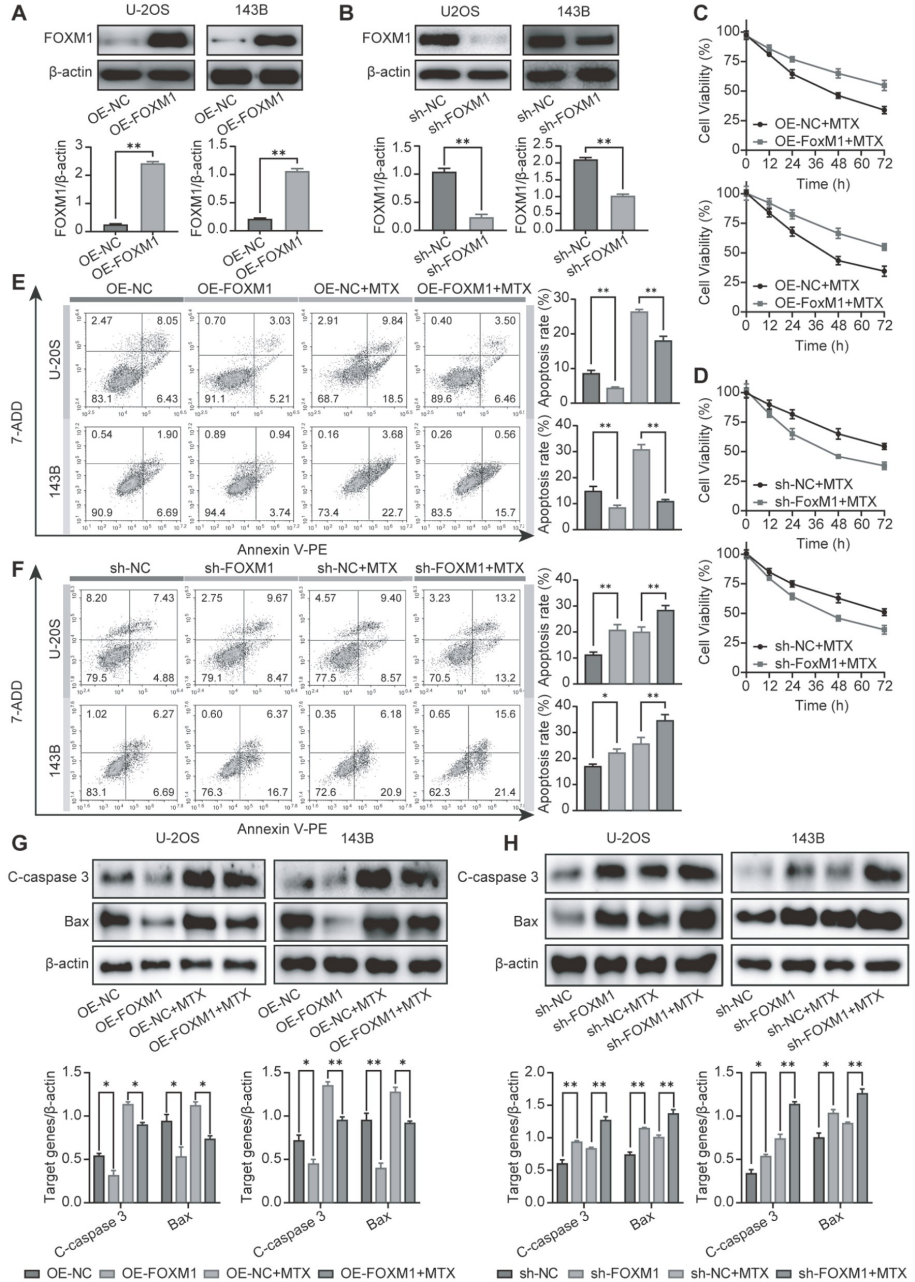
Figure 3 FOXM1 contributes to MTX resistance in OS cells (A,B) The overexpression efficiency of OE-FOXM1 and the knockdown efficiency of sh-FOXM1 were confirmed by western blot analysis in OS cells. (C,D) Cell proliferation assay was carried out after FOXM1-overexpressing cells (C) and FOXM1-knockdown cells (D) were treated with 50 μM MTX for the indicated time. (E,F) After treatment with 50 μM MTX for 24 h, apoptosis was detected by flow cytometry in both FOXM1-overexpressing cells (E) and FOXM1-knockdown cells (F). (G,H) Apoptosis-associated proteins were analyzed by western blot analysis in both FOXM1-overexpressing cells (G) and FOXM1-knockdown cells (H) after treatment with 50 μM MTX for 24 h. n=3, * P<0.05, ** P<0.01.

To better understand the role of FOXM1 in OS resistance to MTX, we generated FOXM1-overexpression and *FOXM1*-knockdown U-2OS and 143B cells via lentiviral transduction. FOXM1 expression was assessed in these cells by western blot analysis. As shown in Figure 3A,B, a significant increase in the expression of FOXM1 was observed in the overexpression cells (OE-FOXM1), while the expression of FOXM1 was significantly reduced in the knockdown clones (sh-FOXM1). Then, the changes in the sensitivity of these cells to MTX were explored using CCK-8 assay. FOXM1 overexpression significantly increased MTX resistance, while *FOXM1* knockdown enhanced MTX sensitivity ( Figure 3C,D). The results of the Annexin-V/PI apoptosis assay indicated that FOXM1 overexpression protected OS cells from MTX-induced apoptosis ( Figure 3E). In contrast, *FOXM1* knockdown increased OS cell apoptosis ( Figure 3F). The western blot analysis results for cleaved caspase-3 and Bax expressions were consistent with the Annexin-V/PI assay results and suggested that FOXM1 overexpression decreased the MTX-induced apoptosis of OS cells ( Figure 3G). Silencing of *FOXM1* with shRNA enhanced apoptosis ( Figure 3H). Taken together, these results indicate that high FOXM1 expression contributes to MTX resistance in OS cells.

### FOXM1 promotes MTX resistance by inducing autophagy in OS cells

**Figure 4:**
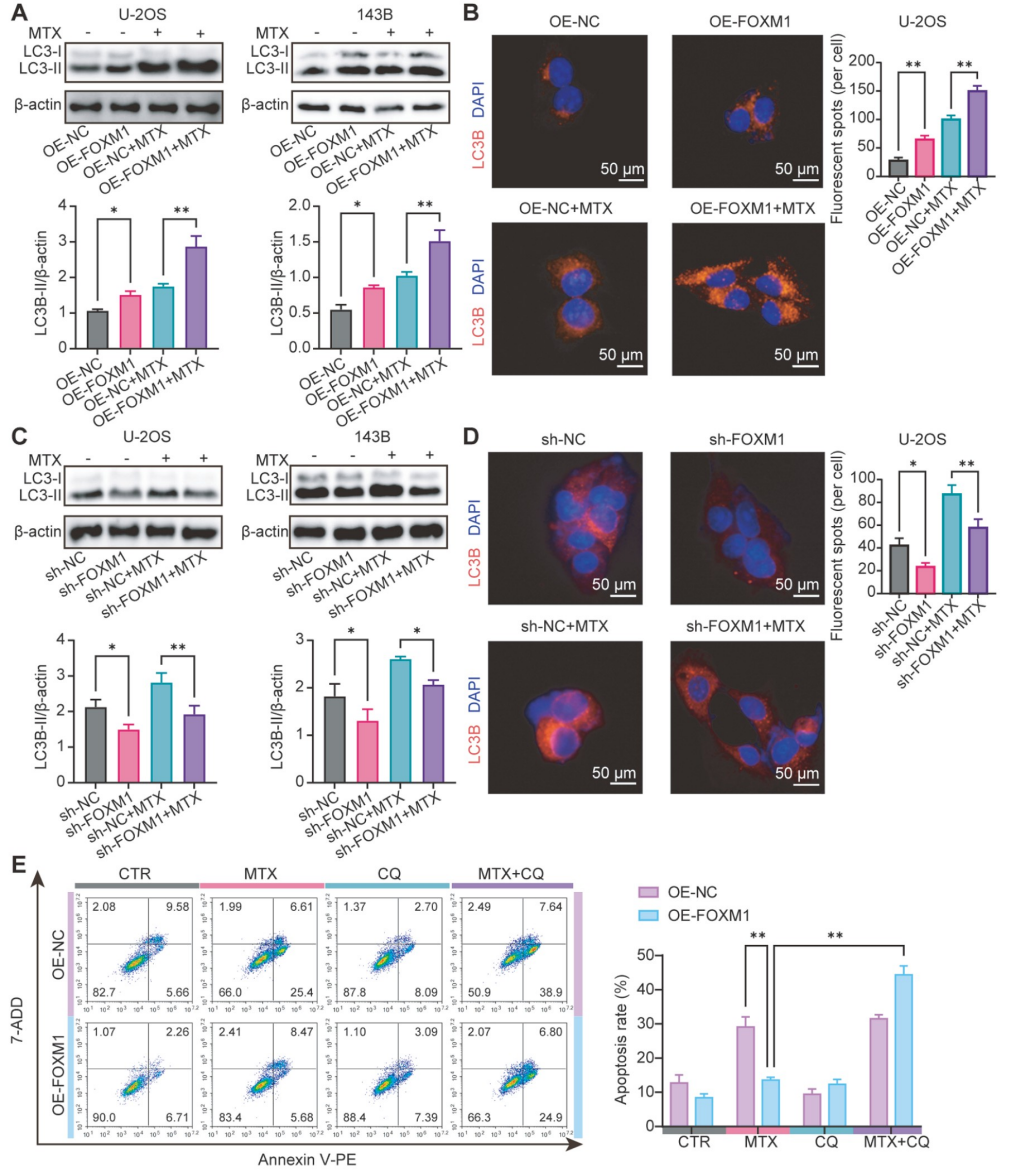
Figure 4 FOXM1 induces MTX resistance by enhancing autophagy in OS cells (A,C) LC3-II protein expression in FOXM1-overexpressing cells (A) or FOXM1-knockdown cells (C) was detected by western blot analysis after treatment with 50 μM MTX for 24 h. (B,D) The expression of LC3B was also detected by immunofluorescence staining in FOXM1-overexpressing cells (B) or FOXM1-knockdown cells (D). Scale bar: 100 μm. After treatment with 50 μM MTX for 24 h in the presence or absence of CQ (20 μM), apoptosis was detected by flow cytometry in FOXM1-overexpressing cells (E). n=3, * P<0.05, ** P<0.01.

Recent findings have indicated that FOXM1-induced autophagy may contribute to chemotherapy resistance in other tumor cells [ 9, 19] . However, whether there is a similar mechanism in OS remains unknown. Thus, we next asked whether autophagy mediates the resistance to MTX induced by FOXM1 in OS cells. As illustrated in Figure 4A, the expression of LC3-II was significantly increased in FOXM1-overexpressing OS cells, especially after treatment with MTX. Immunofluorescence assays further confirmed these results and suggested that autophagy was increased in FOXM1-overexpressing OS cells ( Figure 4B). In contrast, the expression of LC3-II was markedly reduced in *FOXM1*-knockdown OS cells, even in the presence of MTX ( Figure 4C). Similar results were obtained in the immunofluorescence assay ( Figure 4D). To further investigate autophagy’s role in FOXM1-induced MTX resistance in OS cells, the lysosomotropic agent chloroquine (CQ) was used to block autophagy. As expected, the inhibition of autophagy with CQ dramatically reversed the sensitivity of FOXM1-overexpressing OS cells to MTX ( Figure 4E). Collectively, these data demonstrate that autophagy mediates the resistance to MTX induced by FOXM1 in OS cells.

### FOXM1 regulates autophagy via the HMMR/ATG7 pathway

**Figure 5:**
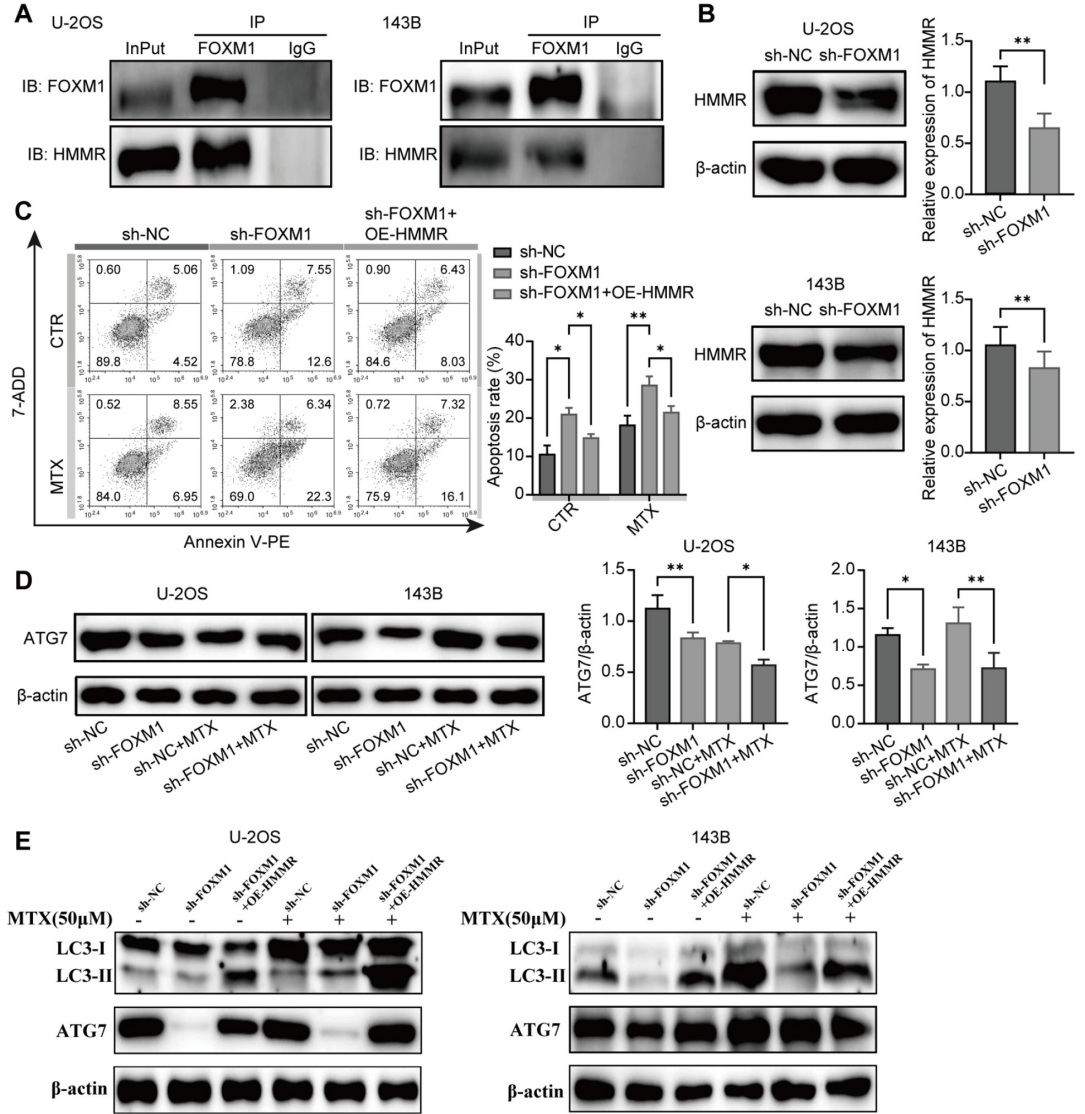
Figure 5 FOXM1 promotes autophagy via interaction with HMMR in OS cells (A) Coimmunoprecipitation was used to assess the interaction between FOXM1 and HMMR in U-2OS and 143B cells with InPut as the whole-cell protein lysate and IgG as the negative control. (B) HMMR protein expression in FOXM1-knockdown U-2OS and 143B cells. β-Actin was used as the loading control. (C) HMMR was overexpressed in FOXM1-knockdown U-2OS cells, and the apoptosis of U-2OS cells treated with or without MTX was detected by flow cytometry. (D) FOXM1 knockdown decreased ATG7 expression as detected by western blot analysis. (E) Overexpression of HMMR restored the expressions of autophagy-related proteins (LC3-II and ATG7) in FOXM1-knockdown OS cells as detected by western blot analysis. n=3, * P<0.05, ** P<0.01.

Recent findings revealed that HMMR is a downstream target of FOXM1 [20]. Moreover, HMMR can increase autophagic lysosome activity and is positively correlated with autophagy [21]. Therefore, we hypothesized that HMMR may participate in regulating autophagy induced by FOXM1. To investigate this possibility, we first performed a coimmunoprecipitation analysis. The results showed that there is a molecular interaction between FOXM1 and HMMR ( Figure 5A). In addition, *FOXM1* knockdown reduced the expression of HMMR, suggesting that FOXM1 is involved in the regulation of HMMR expression ( Figure 5B). Moreover, overexpression of HMMR attenuated the effects of *FOXM1* knockdown on cell apoptosis ( Figure 5C). These results demonstrate that the expression of HMMR is regulated by FOXM1 and may mediate the MTX resistance induced by FOXM1 in OS cells. To further investigate the mechanisms underlying the drug resistance induced by the FOXM1/HMMR pathway, the expression of the autophagy protein Atg7 was detected. As shown in Figure 5D, *FOXM1* knockdown dramatically decreased Atg7 expression in OS cells. In contrast, the results showed that overexpression of HMMR restored the expressions of autophagy-related proteins (LC3-II and ATG7) in *FOXM1*-knockdown OS cells ( Figure 5E). These findings suggest that HMMR can regulate the expression of ATG7 independent of FOXM1. Overall, FOXM1 participates in the regulation of autophagy in OS cells via the HMMR/ATG7 pathway.

### *FOXM1* knockdown enhances the MTX sensitivity of OS in mice

**Figure 6:**
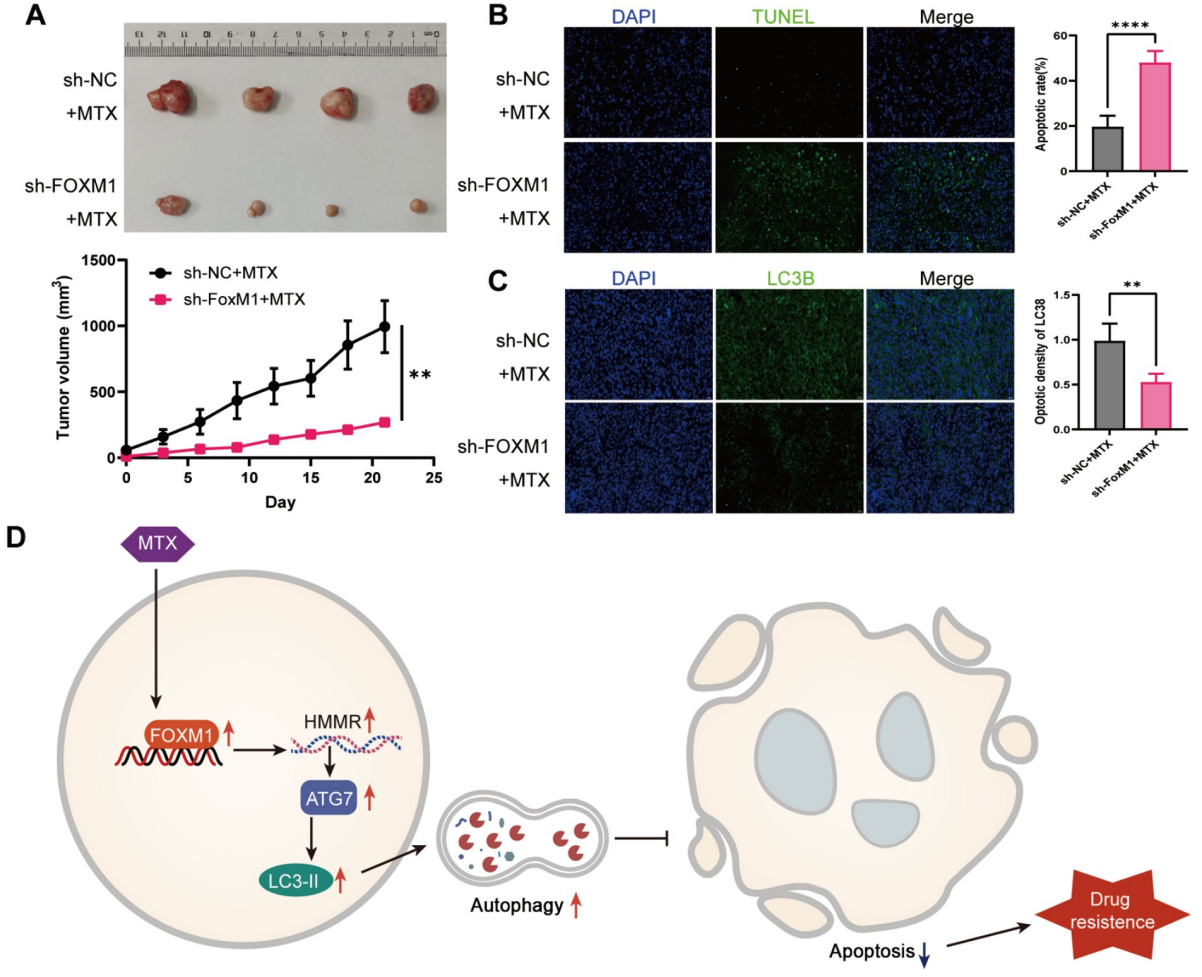
Figure 6 *FOXM1* knockdown enhances autophagy and MTX sensitivity *in vivo* (A) BALB/c nude mice were subcutaneously inoculated with 143B cells with or without FOXM1 knockdown ( n=4). (B) TUNEL staining was used to detect cell apoptosis in the tumor tissues of each group. Scale bar: 20 μm. (C) The protein expression of LC3B in the tumor tissues was detected by immunofluorescence staining. (D) Diagram of the mechanism by which FOXM1 mediates methotrexate resistant cells in osteosarcoma by promoting autophagy. Scale bar: 100 μm. n=3, ** P<0.01, **** P<0.0001.

To determine whether FOXM1 suppression has a similar effect on MTX resistance *in vivo*, 143B cells with or without *FOMX1* silencing were subcutaneously injected into nude mice to establish OS xenograft tumor models. MTX was given intraperitoneally at a dose of 20 mg/kg at the indicated time intervals. As shown in Figure 6A, tumor growth in the *FOXM1* knockdown group was significantly slower than that in the control group. An apoptosis assay using TUNEL staining revealed that *FOXM1* knockdown markedly enhanced OS sensitivity to MTX ( Figure 6B). In addition, the expression of LC3B in the tumor tissues was significantly inhibited in the *FOXM1* knockdown group, as determined by immunofluorescence staining, confirming that FOXM1 could also promote autophagy *in vivo* ( Figure 6C). These data suggest that FOXM1 may serve as a promising target to overcome MTX resistance in OS.

## Discussion

The critical role of FOXM1 in chemotherapy resistance was recently revealed in multiple cancers; however, whether FOXM1 has a similar function in MTX resistance in OS remains unknown. In the present study, we showed that FOXM1 is highly expressed in MTX-resistant OS cells and decreases their sensitivity to MTX by inducing autophagy. Further study revealed that FOXM1 promotes the expression of Atg7 via the induction of HMMR, leading to the upregulation of autophagy and inhibition of apoptosis and ultimately increased resistance to MTX. More importantly, we demonstrated that FOXM1 downregulation could significantly reverse the sensitivity of OS to MTX in mice, providing a promising target for improving OS chemotherapy efficacy.

FOXM1 is a transcription factor of the forkhead family that regulates the expressions of a variety of genes that are essential for numerous cellular processes [22]. For instance, FOXM1 plays an important role in regulating the cell cycle by modulating the transcription of a set of cell cycle‑associated genes [23]. FOXM1 can thereby promote cancer cell proliferation and chemotherapy resistance by facilitating cell cycle progression [24]. FOXM1 can promote and maintain cancer cell stemness in various cancers by regulating the expressions of cancer stem cell phenotype driver genes and interacting with various signaling pathways, including Wnt signaling, the MAPK-ERK pathway, and the PI3K-mTOR pathway [25]. Interestingly, one of the most critical features of cancer stem cells is enhanced resistance to chemotherapeutics [ 6, 26, 27] . Moreover, FOXM1 overexpression protects tumor cells from apoptosis by facilitating DNA repair [28], upregulating antiapoptotic gene expression [29], and regulating microtubule dynamics [30]. Thus, FOXM1 overexpression confers resistance to chemotherapy to cancer cells by counteracting apoptosis [ 31, 32] . In addition to the transcriptional regulation of target genes, the protein–protein interactions of FOXM1 play a critical role in cancer development and therapy [33]. In this study, we demonstrated that FOXM1 not only promoted the expression of the downstream target gene HMMR but also directly interacted with HMMR. The complicated interaction between FOXM1 and HMMR initiates OS autophagy and MTX resistance. However, the detailed role of the FOXM1-HMMR interaction in the induction of autophagy remains to be revealed and deserves further study.

HMMR, a hyaluronan receptor, is a centrosome and microtubule-associated protein that is associated with the cell cycle and mitosis [34]. High expression of HMMR is usually detected in highly proliferative tissues and is associated with poor disease outcomes in a variety of cancers [ 34- 37] . HMMR participates in the maintenance of cancer cell stemness and supports the self-renewal and tumorigenic potential of glioblastoma stem cells [38]. The HMMR signaling pathway endows gastric cancer cells with metastatic capacity by activating AKT signaling [39]. HMMR can also promote lung adenocarcinoma cell expansion in the metastatic niches of hyaluronan-rich microenvironments by enhancing extracellular matrix-mediated signaling [40]. Here, we found that HMMR can promote autophagy by increasing Atg7 expression and inhibiting apoptosis, thus conferring resistance to MTX in OS cells. This finding is inconsistent with previous reports that HMMR could alleviate ER stress by increasing autophagic lysosome activity in hepatocellular carcinoma [21]. Although more detailed mechanistic investigations need to be undertaken, the findings in this study reveal a potential new function of HMMR in cancer progression and provide new targets that may overcome chemotherapy resistance.

Autophagy is a self-degradative cellular process known to counteract different types of cellular stress, including chemotherapy [41]. Autophagy plays an important role in both cancer development and drug resistance, and it is well known to promote tumor cell survival by supplying recycled metabolites for growth [42]. FOXM1 has been reported to promote chemotherapy resistance in an autophagy-dependent manner in a variety of cancers [ 8, 9, 19] . In the present study, our results showed that high FOXM1 expression promotes autophagy via the HMMR/ATG7 pathway in OS. However, the exact underlying mechanism is still not fully understood. Autophagy can suppress the expression of the proapoptotic protein PUMA through autophagic degradation of its promoter [43]. Thus, autophagy decreases cell apoptosis induced by cisplatin, leading to drug resistance in OS [43]. In addition, autophagy may also promote drug resistance via induction of the cancer stem cell phenotype [42].

Drug resistance to chemotherapy remains one of the greatest impediments to cancer treatment. This is especially true for OS, the treatment of which is still largely dependent on chemotherapy. Our results suggest that the FOXM1/HMMR/ATG7 pathway plays an important role in inducing MTX resistance in OS via the promotion of autophagy. Thus, this signaling pathway may serve as a promising target for overcoming MTX resistance. In the last few years, a variety of pharmacological inhibitors of FOXM1 in cancer have been developed [44]. For example, the widely used FOXM1 inhibitor Siomycin A can restore chemotherapeutic sensitivity in a variety of cancer cells [ 11, 45] . However, there is still much work to be done to validate these findings in clinical trials [46]. In addition to FOXM1 inhibition, the use of autophagy inhibitors is another promising approach for overcoming drug resistance [47]. CQ, hydroxychloroquine, bafilomycin A1, and 3-methyladenine are the most commonly used autophagy inhibitors [41]. In this study, we demonstrated that CQ could dramatically reverse the MTX resistance induced by FOXM1 overexpression in OS, suggesting the high potency of autophagy inhibitors in enhancing chemotherapy sensitivity.

In conclusion, our data reveal a new pathway for FOXM1-induced MTX resistance in OS. We show that FOXM1 is highly expressed in OS tissues and MTX-resistant OS cells. FOXM1 overexpression confers MTX resistance to OS cells through the promotion of autophagy via an HMMR/ATG7-dependent pathway. Moreover, silencing of *FOXM1* or inhibiting autophagy reverses drug resistance. These findings provide a promising target to improve the outcomes of chemotherapy in terms of OS.

## References

[1] Cole S, Gianferante DM, Zhu B, Mirabello L (2022). Osteosarcoma: a surveillance, epidemiology, and end results program-based analysis from 1975 to 2017. Cancer.

[2] Bielack SS, Kempf-Bielack B, Delling G, Exner GU, Flege S, Helmke K, Kotz R (2002). Prognostic factors in high-grade osteosarcoma of the extremities or trunk: an analysis of 1,702 patients treated on neoadjuvant cooperative osteosarcoma study group protocols. J Clin Oncol.

[3] Mirabello L, Troisi RJ, Savage SA (2009). Osteosarcoma incidence and survival rates from 1973 to 2004. Cancer.

[4] Beird HC, Bielack SS, Flanagan AM, Gill J, Heymann D, Janeway KA, Livingston JA (2022). Osteosarcoma. Nat Rev Dis Primers.

[5] Gill J, Gorlick R (2021). Advancing therapy for osteosarcoma. Nat Rev Clin Oncol.

[6] Hattinger CM, Patrizio MP, Fantoni L, Casotti C, Riganti C, Serra M (2021). Drug resistance in osteosarcoma: emerging biomarkers, therapeutic targets and treatment strategies. Cancers.

[7] Khan MA, Khan P, Ahmad A, Fatima M, Nasser MW (2023). FOXM1: a small fox that makes more tracks for cancer progression and metastasis. Semin Cancer Biol.

[8] Guo L, Wu Z (2022). FOXM1-mediatedNUF2 expression confers temozolomide resistance to human glioma cells by regulating autophagy via thePI3K/AKT/mTOR signaling pathway. Neuropathology.

[9] Lin J, Wang W, Hu T, Zhu G, Li L, Zhang C, Xu Z (2020). FOXM1 contributes to docetaxel resistance in castration-resistant prostate cancer by inducing AMPK/mTOR-mediated autophagy. Cancer Lett.

[10] Zhu J, Zhao J, Luo C, Zhu Z, Peng X, Zhu X, Lin K (2022). FAT10 promotes chemotherapeutic resistance in pancreatic cancer by inducing epithelial-mesenchymal transition via stabilization of FOXM1 expression. Cell Death Dis.

[11] Hou Y, Dong Z, Zhong W, Yin L, Li X, Kuerban G, Huang H (2022). FOXM1 promotes drug resistance in cervical cancer cells by regulating ABCC5 gene transcription. Biomed Res Int.

[12] Fan CL, Jiang J, Liu HC, Yang D. Forkhead box protein M1 predicts outcome in human osteosarcoma.
*
Int J Clin Exp Med
* 2015, 8: 15563–15568. https://pubmed.ncbi.nlm.nih.gov/26629049/.

[13] Zhu X, Lu K, Cao L, Hu Y, Yin Y, Cai Y (2020). FoxM1 is upregulated in osteosarcoma and inhibition of FoxM1 decreases osteosarcoma cell proliferation, migration, and invasion. Cancer Manag Res.

[14] Kim H, Yoo S, Zhou R, Xu A, Bernitz JM, Yuan Y, Gomes AM,
*et al.* Oncogenic role of SFRP2 in p53-mutant osteosarcoma development via autocrine and paracrine mechanism.
*
Proc Natl Acad Sci USA
* 2018, 115: E11128–E11137. https://doi.org/10.1073/pnas.1814044115.

[15] Chen X, Zhang Q, Dang X, Song T, Wang Y, Yu Z, Zhang S (2021). Targeting the CtBP1-FOXM1 transcriptional complex with small molecules to overcome MDR1-mediated chemoresistance in osteosarcoma cancer stem cells. J Cancer.

[16] Hu K, Xie W, Ni S, Yan S, Tian G, Qi W, Duan Y (2020). Cadmium chloride enhances cisplatin sensitivity in osteosarcoma cells by reducing FOXM1 expression. Oncol Rep.

[17] Yao S, Fan LYN, Lam EWF (2018). The FOXO3-FOXM1 axis: a key cancer drug target and a modulator of cancer drug resistance. Semin Cancer Biol.

[18] Georgiou M, Ntavelou P, Stokes W, Roy R, Maher GJ, Stoilova T, Choo JAMY (2022). ATR and CDK4/6 inhibition target the growth of methotrexate-resistant choriocarcinoma. Oncogene.

[19] Lyu X, Zeng L, Shi J, Ming Z, Li W, Liu B, Chen Y (2022). Essential role for STAT3/FOXM1/ATG7 signaling-dependent autophagy in resistance to Icotinib. J Exp Clin Cancer Res.

[20] Yang D, Ma Y, Zhao P, Ma J, He C (2021). HMMR is a downstream target of FOXM1 in enhancing proliferation and partial epithelial-to-mesenchymal transition of bladder cancer cells. Exp Cell Res.

[21] He L, Li H, Li C, Liu ZK, Lu M, Zhang RY, Wu D (2023). HMMR alleviates endoplasmic reticulum stress by promoting autophagolysosomal activity during endoplasmic reticulum stress-driven hepatocellular carcinoma progression. Cancer Commun.

[22] Bella L, Zona S, Nestal de Moraes G, Lam EWF (2014). FOXM1: a key oncofoetal transcription factor in health and disease. Semin Cancer Biol.

[23] Tan Y, Wang Q, Xie Y, Qiao X, Zhang S, Wang Y, Yang Y (2018). Identification of FOXM1 as a specific marker for triple-negative breast cancer. Int J Oncol.

[24] Arceci A, Bonacci T, Wang X, Stewart K, Damrauer JS, Hoadley KA, Emanuele MJ (2019). FOXM1 deubiquitination by USP21 regulates cell cycle progression and paclitaxel sensitivity in basal-like breast cancer. Cell Rep.

[25] Sher G, Masoodi T, Patil K, Akhtar S, Kuttikrishnan S, Ahmad A, Uddin S (2022). Dysregulated FOXM1 signaling in the regulation of cancer stem cells. Semin Cancer Biol.

[26] Yuan B, Liu Y, Yu X, Yin L, Peng Y, Gao Y, Zhu Q (2018). FOXM1 contributes to taxane resistance by regulating UHRF1-controlled cancer cell stemness. Cell Death Dis.

[27] Modi A, Purohit P, Roy D, Vishnoi JR, Pareek P, Elhence P, Singh P (2022). FOXM1 mediates GDF-15 dependent stemness and intrinsic drug resistance in breast cancer. Mol Biol Rep.

[28] Zhou J, Wang Y, Wang Y, Yin X, He Y, Chen L, Wang W (2014). FOXM1 modulates cisplatin sensitivity by regulating EXO1 in ovarian cancer. PLoS One.

[29] Nestal de Moraes G, Delbue D, Silva KL, Robaina MC, Khongkow P, Gomes AR, Zona S (2015). FOXM1 targets XIAP and Survivin to modulate breast cancer survival and chemoresistance. Cell Signal.

[30] Carr JR, Park HJ, Wang Z, Kiefer MM, Raychaudhuri P (2010). FoxM1 mediates resistance to herceptin and paclitaxel. Cancer Res.

[31] Hu CJ, Wang B, Tang B, Chen B, Xiao YF, Qin Y, Yong X (2015). The FOXM1-induced resistance to oxaliplatin is partially mediated by its novel target gene Mcl-1 in gastric cancer cells. Biochim Biophys Acta.

[32] Xu N, Zhang X, Wang X, Ge H, Wang X, Garfield D, Yang P (2012). FoxM1 mediated resistance to gefitinib in non-small-cell lung cancer cells. Acta Pharmacol Sin.

[33] Gartel AL (2017). FOXM1 in cancer: interactions and vulnerabilities. Cancer Res.

[34] He Z, Mei L, Connell M, Maxwell CA (2020). Hyaluronan mediated motility receptor (HMMR) encodes an evolutionarily conserved homeostasis, mitosis, and meiosis regulator rather than a hyaluronan receptor. Cells.

[35] Zhu H, Tan J, Pan X, Ouyang H, Zhang Z, Li M, Zhao Y (2023). HELLPAR/RRM2 axis related to HMMR as novel prognostic biomarker in gliomas. BMC Cancer.

[36] Li X, Zuo H, Zhang L, Sun Q, Xin Y, Zhang L (2021). Validating HMMR expression and its prognostic significance in lung adenocarcinoma based on data mining and bioinformatics methods. Front Oncol.

[37] Wang C, Thor AD, Moore 2nd DH, Zhao Y, Kerschmann R, Stern R, Watson PH,
*et al.* The overexpression of RHAMM, a hyaluronan-binding protein that regulates ras signaling, correlates with overexpression of mitogen-activated protein kinase and is a significant parameter in breast cancer progression.
*
Clin Cancer Res
* 1998, 4: 567–576. https://pubmed.ncbi.nlm.nih.gov/9533523/.

[38] Tilghman J, Wu H, Sang Y, Shi X, Guerrero-Cazares H, Quinones-Hinojosa A, Eberhart CG (2014). HMMR maintains the stemness and tumorigenicity of glioblastoma stem-like cells. Cancer Res.

[39] Yang M, Chen B, Kong L, Chen X, Ouyang Y, Bai J, Yu D (2022). HMMR promotes peritoneal implantation of gastric cancer by increasing cell-cell interactions. Discov Oncol.

[40] Stevens LE, Cheung WKC, Adua SJ, Arnal-Estapé A, Zhao M, Liu Z, Brewer K (2017). Extracellular matrix receptor expression in subtypes of lung adenocarcinoma potentiates outgrowth of micrometastases. Cancer Res.

[41] Mele L, del Vecchio V, Liccardo D, Prisco C, Schwerdtfeger M, Robinson N, Desiderio V (2020). The role of autophagy in resistance to targeted therapies. Cancer Treat Rev.

[42] Smith AG, Macleod KF (2019). Autophagy, cancer stem cells and drug resistance. J Pathol.

[43] Jiang K, Zhang C, Yu B, Chen B, Liu Z, Hou C, Wang F,
*et al.* Autophagic degradation of FOXO3a represses the expression of PUMA to block cell apoptosis in cisplatin-resistant osteosarcoma cells.
*
Am J Cancer Res
* 2017, 7: 1407–1422. https://pubmed.ncbi.nlm.nih.gov/28744393/.

[44] Kalathil D, John S, Nair AS (2020). FOXM1 and cancer: faulty cellular signaling derails homeostasis. Front Oncol.

[45] Klinhom-On N, Seubwai W, Sawanyawisuth K, Obchoei S, Mahalapbutr P, Wongkham S (2021). FOXM1 inhibitor, Siomycin A, synergizes and restores 5-FU cytotoxicity in human cholangiocarcinoma cell lines via targeting thymidylate synthase. Life Sci.

[46] Liao GB, Li XZ, Zeng S, Liu C, Yang SM, Yang L, Hu CJ (2018). Regulation of the master regulator FOXM1 in cancer. Cell Commun Signal.

[47] Chang H, Zou Z (2020). Targeting autophagy to overcome drug resistance: further developments. J Hematol Oncol.

